# Modular mastery of inflammation: umbilical cord mesenchymal stem cells as a therapeutic frontier

**DOI:** 10.3389/fimmu.2025.1721947

**Published:** 2025-12-19

**Authors:** Li Yin, Chen-yang Sun, Gui-lai Chen, Zhuo Xiang, Bao-quan Hu, Fang Zhou, Qiang Wang

**Affiliations:** 1 The First Clinical Medical College, Shandong University of Traditional Chinese Medicine, Jinan, China; 2 Clinical Medical Research Center, Shandong Second Provincial General Hospital, Jinan, China; 3 Department of Ocardiothoracic Surgery, Southwest Hospital Army of Medical University, Chongqing, China; 4 Hematology Department, The 960th Hospital of The People's Liberation Army (PLA) Joint Logistics Support Force, Jinan, China

**Keywords:** clinical translation, immunomodulation, inflammasome, tissue regeneration, umbilical cord mesenchymal stem cells

## Abstract

Inflammation operates as a dual-edged sword in physiological defense and pathological damage, driving conditions from diabetes to neurodegeneration. Current anti-inflammatory therapies-NSAIDs, corticosteroids, and biologics-face clinical bottlenecks including non-specific toxicity, therapeutic ceiling effects, and drug resistance. Umbilical cord mesenchymal stem cells (UC-MSCs) emerge as a transformative alternative, leveraging three synergistic modules: Immune reprogramming, Inflammasome inhibition, Intercellular communication. Clinical trials demonstrate efficacy in inflammatory bowel disease, COVID-19 ARDS, and graft-versus-host disease. UC-MSCs outperform conventional therapies by multi-pathway modulation and tissue-regenerative capacity, though challenges persist in cell heterogeneity and long-term safety. Future work must standardize dosing protocols and validate scalable production for clinical translation.

## Introduction

Inflammation exhibits a remarkable dual-edged sword characteristic, balancing between physiological defense and pathological damage. As a fundamental mechanism of the body's immune defense, inflammation is critical for maintaining tissue homeostasis and facilitating damage repair (1). However, when this finely regulated immune response becomes dysregulated, excessive inflammation can transform into a pathological driver for various diseases, including diabetes, cardiovascular diseases, and neurodegenerative disorders (2–4). On a basic physiological level, inflammation aids in pathogen clearance and promotes tissue repair; however, its dysregulation may induce chronic inflammatory states, leading to systemic damage. This duality is particularly evident in the progression of liver disease: while inflammation can eliminate damaged cells, its persistent activation drives irreversible fibrotic lesions (5). Therefore, the regulation of inflammation plays a crucial role in maintaining homeostasis and in the prevention and treatment of diseases.

**Figure 1:**
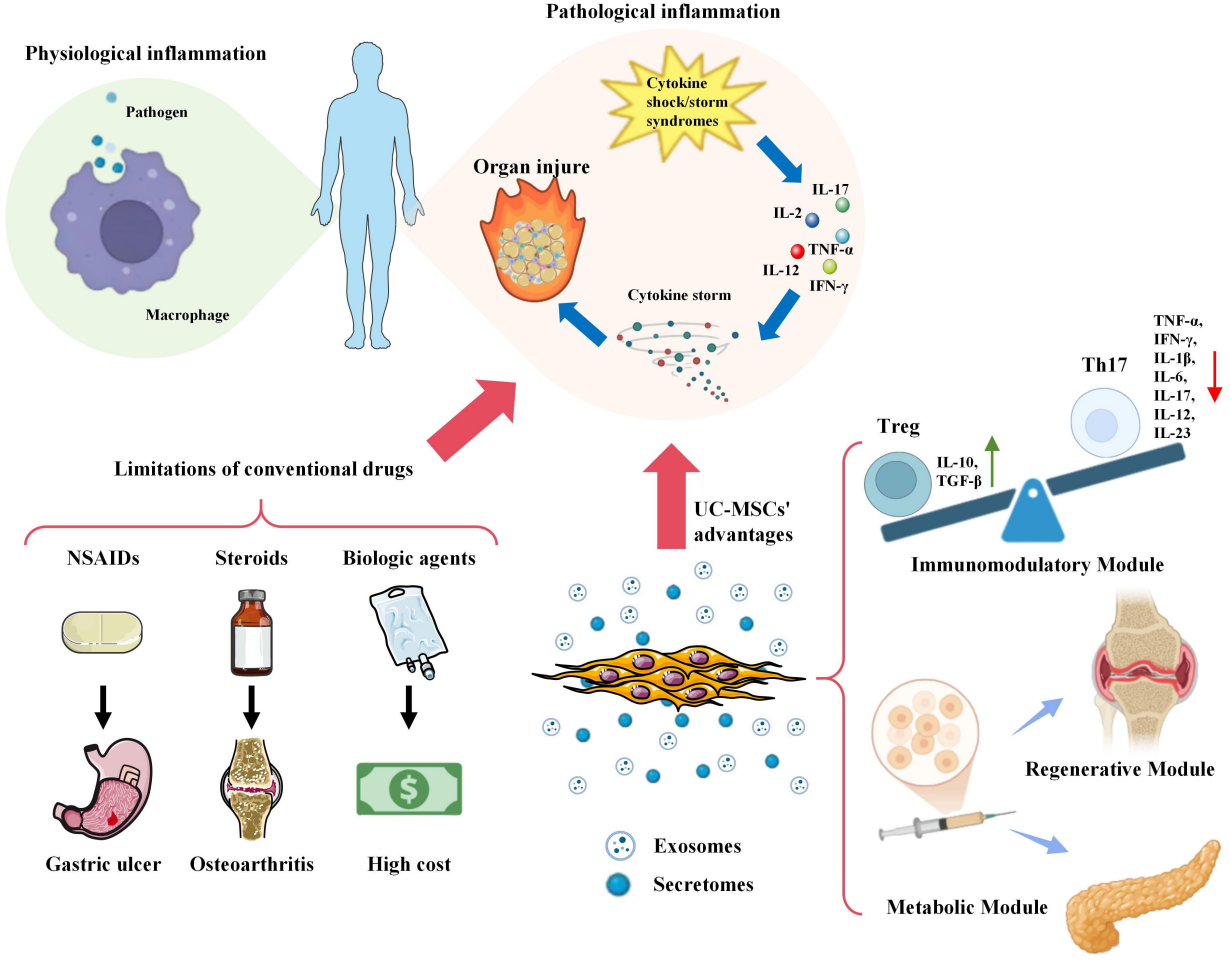
UC-MSCs overcome the limitations of traditional anti-inflammatory therapies through a multi-modal mechanism. Conventional drugs, (such as NSAIDs, steroids, and biologics, are constrained by side effects, cost, and the development of resistance. Human umbilical cord-derived mesenchymal stem cells (UC-MSCs) present a paradigm shift through three core modules: immunomodulation (e.g., exosomal miR-146a-5p/TRAF6, IL-10), tissue regeneration (e.g., differentiation, VEGF/FGF secretion), and metabolic optimization (e.g., Sirt1/Nrf2/HO-1).

Current mainstream anti-inflammatory therapies encounter three clinical bottlenecks (**Figure 1**): Nonsteroidal anti-inflammatory drugs (NSAIDs), by non-selectively inhibiting COX-1/COX-2 enzymes, significantly increase the risk of gastrointestinal ulcers and bleeding (6). Corticosteroids, while potent anti-inflammatories, lead to osteoporosis, immunosuppression, and metabolic disorders with prolonged use (7), and exhibit a therapeutic ceiling phenomenon in treating severe asthma (8). The issue of drug resistance further limits efficacy, with approximately 30% of chronic inflammation patients exhibiting insufficient response to traditional therapies (7). Biologic agents, despite targeting specific inflammatory factors, have limited efficacy in complex microenvironments such as psoriasis (9), and their high cost and potential adverse reactions hinder widespread use (6). Thus, the development of novel, safe, and efficient therapeutic strategies has become an urgent priority in current research.

Umbilical cord-derived mesenchymal stem cells (UC-MSCs), a pluripotent stem cell source derived from Wharton's jelly (rich in extracellular matrix components secreted by mesenchymal stem cells), possess self-renewal, multilineage differentiation, and immunomodulatory properties. In recent years, UC-MSCs have shown significant potential in the treatment of inflammatory diseases. Studies indicate that UC-MSCs not only regulate immune cell function through the secretion of anti-inflammatory factors (such as IL-10) and exosomes, but also exert therapeutic effects through metabolic reprogramming and tissue repair mechanisms (10–12). This review systematically examines the modular mechanisms of UC-MSCs in inflammation regulation, clinical research progress, therapeutic optimization strategies, and future development directions, with the aim of providing a theoretical foundation and practical guidance for the clinical translation of UC-MSCs.

## 2 Modular mechanisms of inflammation regulation by UC-MSCs

**Figure 2:**
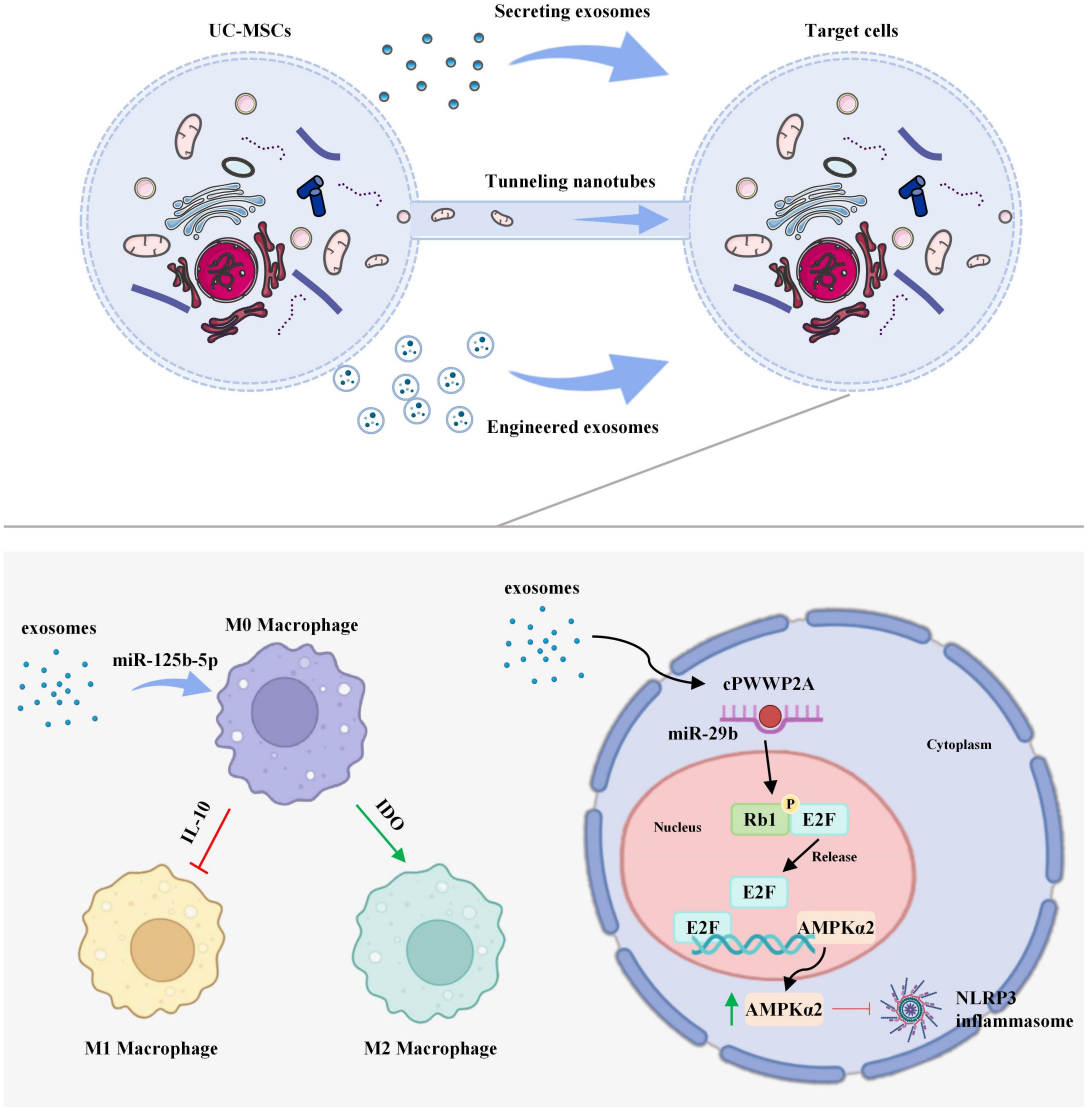
Molecular mechanisms of UC-MSCs' functional modules in inflammatory disease: 1) Immune Reprogramming: UC-MSC-derived exosomes deliver miR-125b-5p to modulate TRAF6 and TGF-β/STAT3 pathways, influencing T-cell and macrophage polarization. 2) Inflammasome Inhibition: Exosomes block NLRP3 assembly via the cPWWP2A-Rb1-AMPKα2 axis. 3) Intercellular Communication: Tunneling nanotubes (TNTs) transfer mitochondria to injured cells, while engineered exosomes deliver therapeutic miR-548e-5p.

The therapeutic efficacy of UC-MSCs in regulating inflammation does not stem from a single molecular pathway, but rather is achieved through a highly evolved, synergistic modular mechanism. Under the guidance of distinct inflammatory microenvironmental signals, UC-MSCs can flexibly activate relatively independent yet interrelated functional modules, including immune cell reprogramming, inflammasome inhibition, and intercellular communication. This enables precise, condition-dependent immunoregulation and tissue repair. The following section will provide a detailed discussion of the operational mechanisms of these three core modules and their synergistic effects (**Figure 2**).

### 2.1 Immune cell reprogramming module

UC-MSCs regulate immune cell phenotype through multiple mechanisms, enabling precise inflammation modulation. In adaptive immunity, UC-MSCs suppress T cell overactivation and promote the differentiation of regulatory T cells (Tregs) by secreting factors such as IL-10 (12). In the balance of Th17/Treg cells, the secretion of TGF-β and chitinase-3-like protein 1 (Chi3l1) by UC-MSCs inhibits STAT3 phosphorylation, thereby blocking Th17 cell differentiation and significantly improving acute graft-versus-host disease (13). In autoimmune diseases such as systemic lupus erythematosus (SLE), UC-MSCs further correct Th17/Treg imbalance through the regulation of miR-125b-5p (14, 15). At the level of innate immunity, UC-MSCs and their exosomes drive macrophage polarization through a dual metabolic-epigenetic regulatory mechanism. For example, by downregulating TRAF1, UC-MSCs promote M2 polarization, alleviating severe asthma (8, 16, 17); and by inhibiting the C3a-C3aR complement signaling pathway, they reverse the abnormal activation of microglial cells (18). This disease-context-specific immune regulation (e.g., inhibiting M1 polarization in inflammatory bowel disease and promoting M2 polarization in rheumatoid arthritis) (19, 20) positions UC-MSCs as a unique immunometabolic checkpoint, with a mechanism that markedly differs from classical immune checkpoint pathways such as PD-1/PD-L1.

### 2.2 Inflammasome inhibition module

UC-MSCs effectively suppress the assembly and activation of the NLRP3 inflammasome through various non-classical pathways, thereby blocking inflammasome-driven cascades. Mechanisms include activation of the cPWWP2A-Rb1-AMPKα2 signaling axis to prevent NLRP3 oligomerization (21). In sepsis models, UC-MSC-derived exosomes inhibit macrophage glycolysis mediated by HIF-1α, reducing the release of inflammatory factors (22). Additionally, their mitochondrial quality control mechanisms promptly clear mitochondrial damage-associated molecular patterns (mitoDAMPs), thereby inhibiting caspase-1 activation (23). In specific disease contexts, UC-MSCs also collaborate with other key signaling pathways to exert anti-inflammatory and antioxidant effects. For instance, in psoriasis, exosomal miR-146a-5p targets TRAF6 to inhibit the NF-κB signaling pathway (9); in interstitial cystitis, UC-MSCs activate the Sirt1/Nrf2/HO-1 pathway to alleviate oxidative stress damage (24). Experimental studies demonstrate that 3D culture technology enhances the NLRP3 suppression efficiency of UC-MSCs by 2.1-fold (25); their exosomes also significantly reduce the expression of MMP13 in osteoarthritis chondrocytes, with a decrease of 2.1-fold (25), thereby functionally confirming the pivotal role of this module in regulating inflammation and matrix degradation.

### 2.3 Intercellular communication module

The intercellular communication module plays a critical role in inflammation regulation primarily through exosome-mediated paracrine signaling and tunneling nanotube (TNT)-mediated direct organelle transfer. In osteoarthritis models, UC-MSC-derived exosomes deliver bioactive substances that effectively upregulate the expression of COL2A1 and SOX-9 in chondrocytes, promoting cartilage matrix synthesis and repair. Simultaneously, secreted growth factors such as VEGF and FGF drive angiogenesis and matrix remodeling (26, 27). Furthermore, TNTs facilitate organelle-level communication, such as transferring functional mitochondria to damaged chondrocytes to restore oxidative phosphorylation, a process resembling prion-like protein transmission patterns (28, 29). In recent years, various technical strategies have further optimized communication efficiency: chitosan scaffolds enhance IL-10 secretion by UC-MSCs threefold, boosting local immunoregulation (30, 31); SEC-purified exosomes significantly improve the specificity of T-cell proliferation inhibition, while avoiding the potential pro-inflammatory risks of non-vesicular components (32); engineered exosomes can deliver molecules such as miR-548e-5p, targeting the TRAF6 pathway to alleviate inflammation in target cells (33). Preclinical studies have validated the potential of these strategies, with 3D culture enhancing exosome curvature and joint targeting. In acute lung injury models, intravenous infusion of UC-MSCs improved rat survival by 40%, providing solid evidence for their clinical translation (1).

In summary, the three functional modules of UC-MSCs-immune cell reprogramming, inflammasome inhibition, and intercellular communication-do not operate in isolation, but rather form a deeply interconnected anti-inflammatory network. For instance, in sepsis models, exosomes (communication module) inhibit HIF-1α-mediated glycolysis (metabolic reprogramming), subsequently suppressing the NLRP3 inflammasome (inhibition module) and promoting macrophage polarization toward the M2 phenotype (reprogramming module) (22). The flexible combination and synergy of these modules enable UC-MSCs to intelligently adapt to diverse disease microenvironments, such as NF-κB inhibition in psoriasis (34) and mitochondrial transfer in osteoarthritis (35), demonstrating unique context-dependent therapeutic advantages. This underscores their core value as a modular immune modulation platform.

## 3 Clinical research on UC-MSCs in inflammation-related diseases

**Figure 3:**
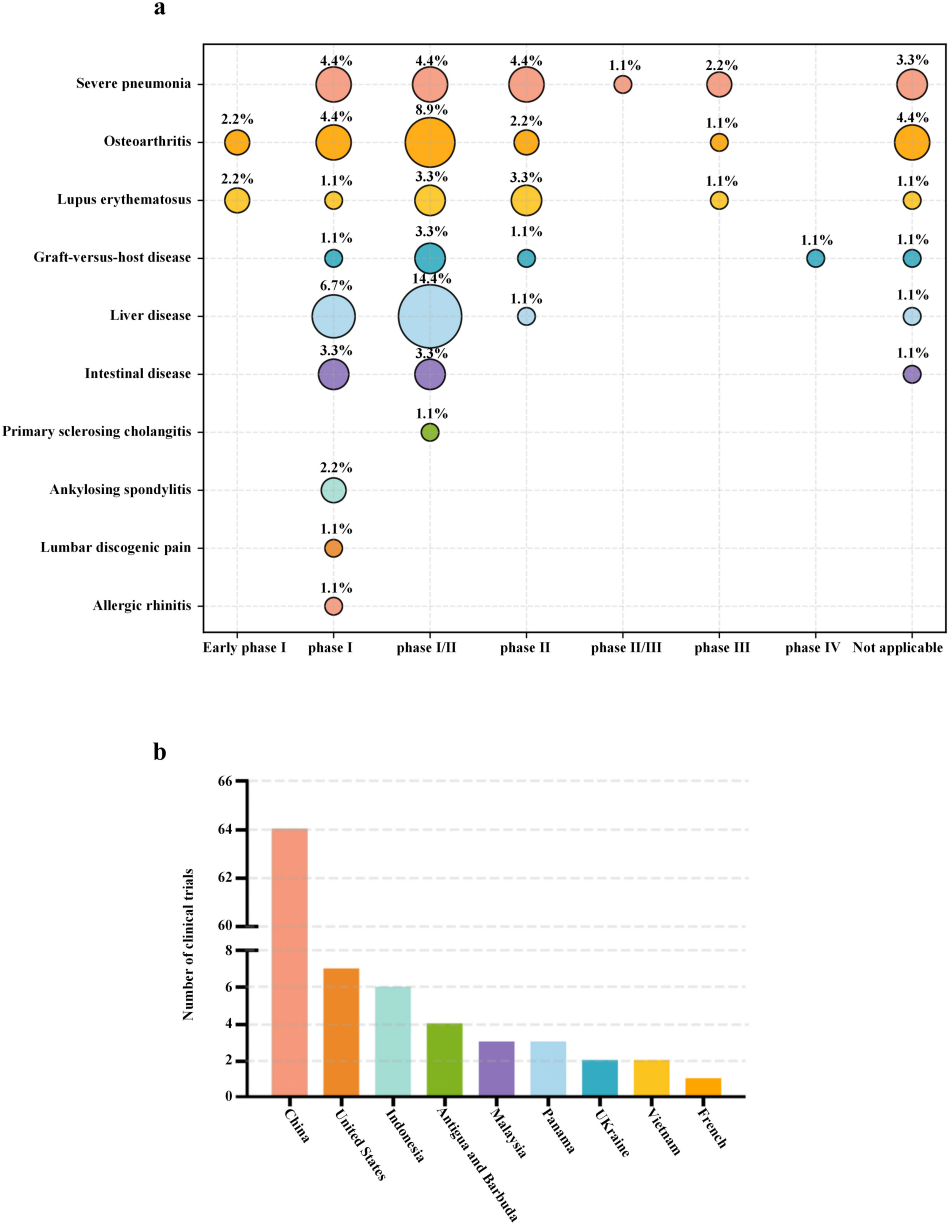
**(a)** Statistics of clinical research on UC-MSCs Therapy for Immune-Related diseases. By statistically analyzing the clinical trials that have been conducted using UC-MSCs for the treatment of immune-related diseases, a conditional diagram was constructed based on the proportion of clinical trials associated with different diseases. Data were obtained using a recurrent search of keywords for medical conditions (appearing in the diagram) associated with UC-MSCs at ClinicalTrials.gov. The search was completed on September 2025. **(b)** Global Statistical Analysis of Clinical Trials on UC-MSCs for the Treatment of Immune-Related Diseases.

Preclinical studies have demonstrated their favorable safety profiles and significant therapeutic efficacy, with numerous clinical trials actively investigating their potential applications in inflammatory disease management (**Table 1**). The U.S. National Institutes of Health ClinicalTrials.gov registry documents nearly one hundred of global clinical trials investigating the use of UC-MSCs in inflammatory diseases (**Figure 3a**). Geographical analysis reveals a concentration of research activity in China, the European Union, and the United States. However, 82.2% of these studies remain in phases I and II, with only 5.6% advancing to phases III and IV (**Figure 3b**). Although preliminary data support the safety profile of UC-MSCs, critical aspects such as optimal dosing regimens, administration routes, and long-term therapeutic efficacy require further systematic evaluation.

**Table 1:** Exploring clinical trials utilizing UC-MSCs for treating inflammation-related diseases (study start dates were from 2020 to 2025).

NCT number	Study title	Phase	Enrollment	Disease	Status	Reference (PMID)
NCT05003947	Safety of Cultured Allogeneic Adult Umbilical Cord Derived Mesenchymal Stem Cell Intravenous Infusion for IBD	I	15	Inflammatory Bowel Diseases	Recruiting	11693327; 19714751; 27882104; 28873511;
NCT05262829	Clinical Study of Human Umbilical Cord Mesenchymal Stem Cells in the Treatment of Moderate and Severe Crohn's Disease	Not Applicable	40	Crohn Disease	Recruiting	
NCT05039411	Safety of Allogeneic Human Umbilical Cord Mesenchymal Stem Cells (UC-MSCs) to Treat Perianal Fistulas Patients With Crohn's Disease	I	7	Perianal Fistula Due to Crohn's Disease; Fistula in Ano	Recruiting	
NCT04939337	A Study of TH-SC01 for Treating Complex Perianal Fistula in Perianal Crohn's Disease.	I	24	Crohn's Disease	Enrolling by invitation	
NCT05682586	UC-MSCs in the Treatment of Severe and Critical COVID-19 Patients	III	60	COVID-19 Pneumonia	Recruiting	
NCT05689008	UC-MSCs in the Treatment of Severe and Critical COVID-19 Patients With Refractory Hypoxia	III	60	COVID-19 Pneumonia	Recruiting	
NCT04273646	Study of Human Umbilical Cord Mesenchymal Stem Cells in the Treatment of Severe COVID-19	Not Applicable	48	COVID-19	Unknown status	
NCT04457609	Administration of Allogenic UC-MSCs as Adjuvant Therapy for Critically-Ill COVID-19 Patients	I	40	COVID-19	Unknown status	
NCT05719012	Efficacy and Safety of Umbilical Cord Mesenchymal Stem Cells in the Treatment of Long COVID-19	II	70	Long COVID-19	Not yet recruiting	
NCT04355728	Use of UC-MSCs for COVID-19 Patients	I/II	24	Corona Virus Infection; COVID-19	Completed	
NCT05501418	Evaluate the Safety and Efficacy of Allogeneic Umbilical Cord Mesenchymal Stem Cells in Patients With COVID-19 (UMSC01)	I/II	75	COVID-19 Infection	Active, not recruiting	
NCT04288102	Treatment With Human Umbilical Cord-derived Mesenchymal Stem Cells for Severe Corona Virus Disease 2019 (COVID-19)	II	100	Corona Virus Disease 2019 (COVID-19)	Completed	
NCT04461925	Treatment of Coronavirus COVID-19 Pneumonia (Pathogen SARS-CoV-2) With Cryopreserved Allogeneic P_MMSCs and UC-MMSCs	I/II	30	COVID-19 Pneumonia	Unknown status	
NCT05286255	Mesenchymal Stromal Cells for COVID-19 and Viral Pneumonias (SAMPSON-1)	I	10	COVID-19 Pneumonia Viral Pneumonia	Not yet recruiting	
NCT04269525	Umbilical Cord(UC)-Derived Mesenchymal Stem Cells(MSCs) Treatment for the 2019-novel Coronavirus(nCOV) Pneumonia	II	16	Pneumonia, Viral	Unknown status	
NCT04573270	Mesenchymal Stem Cells for the Treatment of COVID-19	I	40	COVID-19	Completed	
NCT04429763	Safety and Efficacy of Mesenchymal Stem Cells in the Management of Severe COVID-19 Pneumonia (CELMA)	II	30	COVID-19	Unknown status	
NCT04461925	Treatment of Coronavirus COVID-19 Pneumonia (Pathogen SARS-CoV-2) With Cryopreserved Allogeneic P_MMSCs and UC-MMSCs	I/II	30	COVID-19 Pneumonia	Unknown status	
NCT04490486	Umbilical Cord Tissue (UC) Derived Mesenchymal Stem Cells (MSCs) Versus Placebo to Treat Acute Pulmonary Inflammation Due to COVID-19 (COVID-19)	I	0	COVID-19	Withdrawn	
NCT05132972	Allogenic UCMSCs as Adjuvant Therapy for Severe COVID-19 Patients (UCMSC)	II/III	42	COVID-19	Recruiting	
NCT04273646	Study of Human Umbilical Cord Mesenchymal Stem Cells in the Treatment of Severe COVID-19	Not Applicable	48	COVID-19	Unknown status	
NCT05160831	Human Umbilical Cord Mesenchymal Stem Cells in the Treatment of Knee Osteoarthritis	Not Applicable	50	Knee Osteoarthritis	Not yet recruiting	
NCT05147675	Safety of Cultured Allogeneic Adult Umbilical Cord Derived Mesenchymal Stem Cells for OA	I	20	Osteoarthritis	Recruiting	29786336; 30522806; 31639063; 27217345
NCT04971980	Safety and Efficacy Study of Human Umbilical Cord-Derived Mesenchymal Stem Cells(BC-U001) for Rheumatoid Arthritis	I/II	9	Rheumatoid Arthritis	Recruiting	
NCT05003934	Safety of Cultured Allogeneic Adult Umbilical Cord Derived Mesenchymal Stem Cell Intravenous Infusion for RA	I	20	Rheumatoid Arthritis	Recruiting	18490431; 30211382; 31908418; 30551438
NCT06082440	Study on the Safety and Tolerance of Mesenchymal Stem Cells Mediated by Arthroscopy in Patients With Osteoarthritis	Early I	18	Osteoarthritis	Recruiting	
NCT05016011	Efficacy of Allogeneic UCMSCs for Treating Large Defects Knee Injury	II	50	Osteoarthritis, Knee	Unknown status	
NCT05579665	Effectiveness of PRP, Conditioned Medium UC-MSCs Secretome and Hyaluronic Acid for the Treatment of Knee Osteoarthritis	I/II	45	Knee Osteoarthritis	Active, not recruiting	23104611; 25624776; 27384560; 23270598; 35298024; 22534958.
NCT05151133	Clinical Study of Allergic Rhinitis Therapy by Stem Cells	I	18	Allergic rhinitis	Recruiting	
NCT05531266	Umbilical Cord Mesenchymal Stem Cells as First-line Treatment for Patients With Acute Graft Versus Host Disease	Not Applicable	182	aGVHD	Recruiting	
NCT05152160	Umbilical Cord Mesenchymal Stem Cells in the Treatment of Moderate/​Severe Chronic Graft-versus-host Disease	I/II	10	Graft Vs Host Disease	Recruiting	
NCT06149832	Treatment of Oral Chronic Graft-versus-host Disease With Human Umbilical Cord Mesenchymal Stem Cell Dressing	IV	38	GVHD	Recruiting	
NCT05984303	Human Umbilical Cord-derived Mesenchymal Stem Cells for Decompensated Cirrhosis (MSC-DLC-1b)	I	6	Decompensated Cirrhosis	Not yet recruiting	
NCT05106972	Umbilical Cord Mesenchymal Stem Cell Transplantation for Decompensated Hepatitis B Cirrhosis	Not Applicable	30	Liver Cirrhosis	Recruiting	
NCT05227846	Human Umbilical Cord-derived Mesenchymal Stem Cells for Decompensated Cirrhosis (MSC-DLC-1)	I	12	Decompensated Cirrhosis	Recruiting	
NCT05121870	Treatment With Human Umbilical Cord-derived Mesenchymal Stem Cells for Decompensated Cirrhosis	II	240	Decompensated Cirrhosis	Recruiting	
NCT05224960	Human Umbilical Cord-derived Mesenchymal Stem Cells for Decompensated Cirrhosis (MSC-DLC-2)	II	240	Decompensated Cirrhosis	Not yet recruiting	
NCT05331872	Umbilical Cord-derived Mesenchymal Stem Cell Infusion in the Management of Adult Liver Cirrhosis	I	20	Liver Cirrhosis	Recruiting	
NCT05155657	Study of Decompensated Alcoholic Cirrhosis Treatment by Stem Cells	I	36	Alcoholic Cirrhosis	Recruiting	
NCT05948982	Safety of Umbilical Cord Mesenchymal Stem Cells (UC-MSC) in Patients With Decompensated Hepatitis B Cirrhosis	I/II	18	Decompensated Liver Cirrhosis	Not yet recruiting	
NCT05507762	Study of Human Umbilical Cord Mesenchymal Stem Cell in Patients With Cirrhosis Due to Hepatitis B (Compensation Stage)	I/II	20	Cirrhosis Due to Hepatitis B	Recruiting	
NCT06485648	Mesenchymal Stem Cell Transfusion for the Treatment of Refractory Lupus Nephritis (LN)	Early I	96	Lupus Nephritis	Not yet recruiting	
NCT05018858	Safety of Cultured Allogeneic Adult Umbilical Cord Derived Mesenchymal Stem Cell Intravenous Infusion for Lupus	I	15	Lupus	Recruiting	24661633; 24388428; 20506343.
NCT05631717	The Study of Comparing the Efficacy and Safety of Human Umbilical Cord MSCs and Low-dose IL-2 in the Treatment of LN	III	40	Systemic Lupus Erythematosus Lupus Nephritis	Recruiting	32355793; 30428931; 28808198; 28724445; 28217916; 28129605; 26537898; 25611801; 21617158; 21837432; 20650877; 20517294.
NCT05962762	Safety and Tolerance of Umbilical Cord Mesenchymal Stem Cells (UC-MSC) in Patients With Ankylosing Spondylitis	I	9	Ankylosing Spondylitis	Not yet recruiting	

### 3.1 Inflammatory bowel disease

Inflammatory bowel disease (IBD), including ulcerative colitis (UC) and Crohn's disease (CD), is an idiopathic chronic gastrointestinal inflammation (36). UC is typically confined to the rectum and colon, while CD primarily affects the terminal ileum and colon (37). Multiple clinical studies have validated the safety and efficacy of mesenchymal stem cells (MSCs) derived from bone marrow, umbilical cord, and adipose tissue in treating IBD through intravenous or local injection (36). Zhang et al. investigated the preliminary efficacy and safety of UC-MSCs infusion in treating CD (NCT02445547), enrolling 82 patients who had been undergoing corticosteroid maintenance therapy for more than six months. Forty-one patients received UC-MSCs transplantation (administering four doses of 1×10^6^ cells/kg via peripheral intravenous infusion, once a week). Follow-up results showed that the UC-MSCs group significantly improved the disease severity after corticosteroid treatment, with decreases in CDAI, HBI, and corticosteroid dose of 62.5 ± 23.2, 3.4 ± 1.2, and 4.2 ± 0.84 mg/day, respectively, while the control group exhibited reductions of 23.6 ± 12.4, 1.2 ± 0.58, and 1.2 ± 0.35 mg/day, respectively (UC-MSCs vs. control group, *p* < 0.01, *p* < 0.05, *p* < 0.05). Four patients developed fever after cell infusion, but no serious adverse events were observed (38). A Phase I/II study conducted by Hu et al. evaluated the effects of UC-MSCs infusion via peripheral blood and mesenteric artery for the treatment of severe UC (NCT01221428). Seventy patients were divided into two groups, with 34 receiving UC-MSCs treatment and 36 receiving saline. After one month, 30 patients in the UC-MSCs group showed good responses, significantly improving diffuse and deep ulcers and inflammatory mucosa. During follow-up, the Mayo score and histological scores in the UC-MSCs group were significantly lower than those before treatment and in the control group, with a significant increase in the median IBDQ score (*p* < 0.05). No significant adverse reactions were observed in the MSC group, and no chronic side effects or sequelae were noted. These results indicate that MSC transplantation is a safe and effective treatment, capable of alleviating diffuse and deep lesions as well as mucosal inflammation (39).

### 3.2 Cirrhosis

Cirrhosis is a chronic disease caused by liver inflammation and fibrosis. Common etiologies include viral hepatitis, steatohepatitis, autoimmune hepatitis, sclerosing cholangitis, and Wilson's disease (40). Additionally, alcohol consumption can lead to and exacerbate cirrhosis and other chronic liver diseases. Autoimmune hepatitis (AIH) is a complex autoimmune disease that affects individuals of all ages and genders. Its serological features include elevated transaminase levels, increased polyclonal immunoglobulin G (IgG), and the presence of characteristic autoantibodies (41). The primary treatment currently consists of a combination of corticosteroids and immunosuppressive agents. In recent years, MSC therapy has emerged as a new option for treating end-stage liver disease. Several clinical studies have shown that infusion of autologous bone marrow-derived mesenchymal stem cells (BM-MSCs) can significantly improve liver function in patients with cirrhosis (42, 43). Particularly in the treatment of liver failure, BM-MSCs have been demonstrated to have good safety and feasibility (44, 45). Furthermore, studies have indicated that autologous BM-MSCs not only improve liver function in patients with alcoholic cirrhosis but also effectively alleviate histological fibrosis (46). Moreover, allogeneic MSCs (e.g., allogeneic BM-MSCs, UC-MSCs, and umbilical cord blood-derived MSCs) have shown good safety and efficacy in treating cirrhosis induced by autoimmune diseases (47). Among these, UC-MSCs therapy has been confirmed to significantly improve liver function in patients with decompensated cirrhosis and primary biliary cirrhosis, as well as improve survival rates in patients with acute chronic liver failure (47, 48). A prospective, open-label, randomized controlled trial assessed the effects of UC-MSCs therapy on hepatitis B virus-related non-fully compensated liver cirrhosis (NCT01220492). The study enrolled 219 patients (108 in the treatment group, 111 in the control group). The treatment group received three UC-MSCs infusions at 4-week intervals, alongside standard treatment, while the control group received only standard treatment. The results showed that, during the 13 to 75 months of follow-up, the treatment group had a significantly higher overall survival rate compared to the control group, with no significant difference in survival rates without hepatocellular carcinoma at 75 months between the two groups. The treatment group showed significant improvement in liver function markers (such as serum albumin, prothrombin activity, cholinesterase, and total bilirubin levels) within 48 weeks, with no obvious adverse effects or treatment-related complications (49). Another study in decompensated cirrhosis patients explored the immunomodulatory effects of UC-MSCs. It found that, after treatment, levels of IL-6 and tumor necrosis factor-α (TNF-α) significantly decreased, while IL-10 levels significantly increased. Additionally, T4 and Treg cells significantly increased within 2 to 4 weeks, while T8 cells and B cells markedly decreased. After 8 to 12 weeks of treatment, liver function improved, with significant improvements in aspartate aminotransferase, albumin, total bilirubin, and prothrombin time (50). Furthermore, another study evaluated the impact of UC-MSCs on immune function and prognosis in patients with hepatitis B-induced cirrhosis. The study included 65 patients, with the treatment group receiving intra-arterial or portal venous injections of 4×10^8^ UC-MSCs, while the control group received traditional treatment. The results showed that the treatment group had a significant reduction in IL-6, significantly increased IL-10 and TGF-β levels, and a notable increase in CD4+T and Treg cells, while CD8+T cells and B cells significantly decreased (all *p* < 0.001). Compared to the control group, UC-MSCs therapy significantly alleviated liver failure (6.45% vs 14.71%, *p* = 0.017) (51).

### 3.3 COVID-19

COVID-19, caused by the severe acute respiratory syndrome coronavirus 2 (SARS-CoV-2), is a global infectious disease (52, 53). While most mild cases exhibit self-limiting characteristics, severe patients may rapidly progress to hypoxemia, acute respiratory distress syndrome (ARDS), septic shock, and multiple organ dysfunction syndrome (MODS), ultimately leading to death (54). Recent studies indicate that the pathophysiology of COVID-19-related ARDS is closely associated with excessive inflammatory responses, and UC-MSCs have shown significant anti-inflammatory effects by modulating the expression of pro-inflammatory mediators such as TNF-α (55). Notably, UC-MSCs have been shown to have protective effects in acute lung injury models induced by H5N1 influenza virus (56). Several clinical studies provide key evidence supporting the efficacy and safety of UC-MSCs therapy for COVID-19. In a double-blind, phase I/II randomized controlled trial (NCT04355728), 24 patients with ARDS were randomly assigned to receive UC-MSCs treatment (100 ± 20×10^6^ cells, intravenous infusion on days 0 and 3) or standard treatment. The results revealed that no severe adverse events, such as cardiac arrest, occurred within 6- or 24-hours post-infusion in the treatment group, and inflammatory markers significantly decreased by day 6 (*p* < 0.05). More importantly, the survival rate at 31 days in the treatment group was significantly higher than that in the control group (91% vs. 42%, *p* = 0.015) (57). Another study, compared combination therapy (1×10^6^ cells/kg UC-MSCs + standard treatment) with standard treatment alone. The treatment group showed a 15% improvement in the oxygenation index (*p* = 0.021), with a 40% ± 5% reduction in pulmonary inflammatory infiltration area, and CT imaging showed a significant recovery trend in lesion density (58). A dose-exploration study for severe patients found that a four-round infusion regimen (1×10^8^ cells per infusion, with 24-hour intervals) significantly improved the oxygenation index (PaO2/FiO2 increased by 60 mmHg), and reduced the mortality rate from 45.4% in historical controls to 6.25% (59). In a larger-scale randomized double-blind trial (NCT04288102), 101 patients with lung injury were allocated in a 2:1 ratio to the UC-MSCs group (4×10^7^ cells per infusion) or the placebo group. At 28 days of follow-up, the treatment group showed a 13.31% reduction in total lung lesion volume (*p* = 0.080), a 15.45% decrease in the proportion of solid lesions (*p* = 0.043), and a 27-meter increase in 6-minute walking distance (*p* = 0.057). Notably, 1-year follow-up data revealed that 78% of patients in the UC-MSCs group had complete recovery of lung CT, significantly higher than the 42% in the placebo group (60, 61). A multicenter study from Turkey (n = 210) further confirmed that UC-MSCs therapy improved survival rates by 47.5% in severe patients (OR = 1.475, 95% CI: 1.193-1.824, *p* < 0.001), particularly when intervention was implemented before mechanical ventilation, significantly improving prognosis (62). A single-center randomized controlled trial in Huangshi, China (n = 58) showed that the conversion rate to critical illness in the UC-MSCs treatment group was 0%, with a 10.34% reduction in 28-day mortality compared to the control group (*p* = 0.014), and significant decreases in inflammatory markers (CRP, IL-6) were observed 72 hours after infusion (*p* < 0.01) (63). Current clinical evidence suggests that UC-MSCs demonstrate unique therapeutic advantages in the treatment of COVID-19 through multiple mechanisms, including modulation of cytokine storms, promotion of alveolar epithelial repair, and improvement of microcirculation. Its safety profile has been validated through numerous randomized controlled trials, with no significant difference in the incidence of major adverse events compared to placebo groups. Future studies need to further clarify the optimal treatment timing, administration routes, and dose-response relationships to optimize clinical efficacy.

### 3.4 Arthritis

Osteoarthritis (OA) is a chronic disease characterized by degeneration, destruction, and osteogenesis of joint cartilage, often accompanied by chronic pain, a decline in quality of life, and, in severe cases, mortality (64, 65). As the disease progresses, the medical costs and societal burden due to early loss of labor in OA patients continue to rise (66). Currently, there is a lack of effective therapeutic drugs for OA, with existing treatments primarily providing only short-term relief of clinical symptoms. UC-MSCs with their superior cloning, proliferation, and migration abilities, as well as an inflammation-dependent homing mechanism related to tissue repair, have shown potential in promoting the secretion of cartilage-forming factors, thus demonstrating therapeutic potential for OA (67). A randomized, double-blind, controlled Phase I/II trial was conducted to evaluate the safety and efficacy of single-dose or repeated-dose intra-articular UC-MSCs compared with repeated-dose intra-articular hyaluronic acid (HA) in knee OA patients (NCT02580695). The target population included individuals aged 40-65, with 40 patients screened and 29 patients randomly assigned in a 1:1:1 ratio via an electronic data entry system. The three study groups consisted of: a control group receiving HA intra-articular injections at baseline and 6 months (n = 8); a single-dose (20×10^6^ cells) UC-MSCs group at baseline (MSC-1, n = 9); and a repeated-dose UC-MSCs group (20×10^6^×2) at baseline and 6 months (MSC-2, n = 9). UC-MSCs injections contained 20×10^6^ UC-MSCs in 3cc physiological saline with 5% AB plasma, HA injections contained 3cc Durolan, and placebo injections contained 5% AB plasma in 3cc physiological saline. The results revealed no severe adverse events during the 12-month follow-up, with clinical scores and MRI evaluations assessed. Patients treated with UC-MSCs experienced significant improvements in pain and function (*p* = 0.001). At 12 months, the arthritis index (WOMAC-A; pain scale) in the MSC-2 group (1.1 ± 1.3) showed a significant reduction in pain levels compared to the control group (4.3 ± 3.5; *p* = 0.04). Additionally, at 12 months, the visual analog scale for pain in the MSC-2 group was significantly lower than in the HA group (2.4 ± 2.1 vs. 22.1 ± 9.8, *p* = 0.03). For the total WOMAC score, the MSC-2 group had a lower score than the HA group at 12 months (4.2 ± 3.9 vs. 15.2 ± 11, *p* = 0.05). No differences in MRI scores were observed between the two groups. In this Phase I/II trial, repeated UC-MSCs treatment for knee OA was shown to be safe and superior to the control group at the 1-year follow-up (68).

### 3.5 Graft-versus-host disease

Graft-versus-host disease (GVHD) is a major complication of allogeneic hematopoietic stem cell transplantation (allo-HSCT), and its prevention and treatment strategies remain a critical focus of clinical research. The incidence of acute GVHD (aGVHD) in allo-HSCT patients is approximately 40%, with the risk of occurrence significantly associated with factors such as donor origin, stem cell type, and prophylactic regimen (69). This pathological process begins with the recognition of host tissue compatibility antigens by donor T cells, triggering a cascade inflammatory response that leads to damage in target organs such as the skin, gastrointestinal tract, and liver. Typical manifestations include maculopapular rashes, bloody diarrhea, and cholestatic jaundice (70, 71). Notably, around 20% of aGVHD cases may progress to chronic GVHD or overlap syndrome, significantly impacting long-term prognosis (71). MSCs have emerged as a promising therapeutic approach for GVHD due to their unique immunomodulatory properties. Basic research indicates that UC-MSCs significantly reduce the incidence of chronic GVHD (cGVHD) and promote stable graft engraftment by inhibiting interferon-γ (IFN-γ) secretion and modulating T-cell toxicity (72). Clinical translational studies further confirm this mechanism. In a cohort of 52 patients with severe aGVHD (median age 12.5 years), after receiving a median of 4 UC-MSCs infusions (4.73×10^6^ cells/kg ± 1.30×10^6^ cells/kg), the overall response rate at day 28 reached 63.5%. Importantly, the complete remission rate in pediatric patients (48.6%) was significantly higher than in the adult group (11.8%), with survival benefits showing a statistically significant difference (180-day overall survival: 48.6% vs 17.6%, *p* = 0.038) (73). A Phase II clinical trial on steroid-refractory aGVHD (n = 54) demonstrated that after 28 days of UC-MSCs treatment, the overall response rate (ORR) reached 59.3% (32/54), with 24 patients achieving complete remission (CR) and 8 achieving partial remission (PR). Clinical benefits were significantly better in low-grade GVHD patients (grades I-II) compared to high-grade cases (grades III-IV) (*p* < 0.05) (74). In exploring preventive strategies, a randomized controlled trial including 148 haploidentical transplant patients (Chinese Clinical Trial Registry: ChiCTR-IIR-16007806) demonstrated that a UC-MSCs prophylactic regimen (1×10^6^ cells/kg every two weeks, starting 45 days post-transplant, for a total of four doses) reduced the incidence of grade II-IV aGVHD from 32.4% to 14.9% (*p* = 0.01), while significantly lowering the cumulative incidence of severe cGVHD (2-year: 5.4% vs 17.4%, HR = 0.29, *p* = 0.03), without increasing the risk of leukemia relapse (*p* = 0.68) (75). Mechanistic studies have revealed that UC-MSCs exert their therapeutic effects through dual pathways: ① secretion of the CXCL1 chemokine to recruit myeloid-derived suppressor cells (MDSCs), thereby establishing an immune-tolerant microenvironment (76); ② exosome-mediated delivery of miR-223 to suppress the expression of ICAM-1 in lymphatic endothelial cells, thereby reducing inflammatory cell infiltration (77). Safety assessments indicate that the combination of UC-MSCs infusion (1×10^6^ cells/kg) in pediatric patients with severe aplastic anemia undergoing allo-HSCT did not increase the risk of graft failure. The median times to neutrophil and platelet engraftment were 14 days and 25 days, respectively, and the incidence of grade III-IV aGVHD was controlled below 20% (78). Existing evidence indicates that UC-MSCs demonstrate multi-dimensional advantages in the prevention and treatment of GVHD: ① regulation of the Th1/Th2 balance and inhibition of excessive immune responses (79); ② promotion of tissue repair and improvement of transplantation-related complications such as hemorrhagic cystitis (incidence reduced by 55.2%, *p* = 0.02) (75); ③ a better dose-response relationship in pediatric patient populations (73).

### 3.6 Lupus

Lupus is a chronic autoimmune disease characterized by the immune system abnormally attacking the body's own healthy tissues, leading to inflammation and organ damage. This disease can affect multiple organ systems, including the skin, joints, kidneys, heart, lungs, blood system, and brain. The clinical types of lupus include SLE, discoid lupus erythematosus, drug-induced lupus erythematosus, and neonatal lupus erythematosus. Among these, SLE is the most common and severe form, accounting for approximately 70% of all cases. Due to its involvement of multiple organ systems, complex disease progression, and difficulty in treatment, SLE holds a prominent position in clinical research (80). In the modulation of abnormal immune responses in SLE patients, both UC-MSCs and their exosomes can inhibit the activity of CD4+ T cells, promote the generation of Th17 cells, and increase the production of IL-17 and TGF-β1. However, only UC-MSCs can inhibit the proliferation of CD19+ B cells and promoting the production of IFN-γ and IL-4, without significantly affecting the expression of Th1, Th2, Tfh, Treg, and IL-10 (*p*>0.05) (81). Long-term follow-up studies have shown that UC-MSCs transplantation demonstrates good long-term safety in the treatment of refractory SLE (82). A long-term follow-up study involving 81 patients with severe SLE (NCT00698191 and NCT01741857) revealed that after allogeneic UC-MSCs transplantation, the 5-year overall survival rate was 84%, with 34% of patients achieving disease remission. Additionally, SLE disease activity index, serum albumin, and complement C3 levels continuously improved, confirming both its safety and long-term efficacy (83). These findings provide strong support for the clinical application of UC-MSCs in SLE and suggest that they may become an effective therapeutic approach for SLE treatment.

### 3.7 Rheumatoid arthritis

Rheumatoid arthritis (RA) is a chronic inflammatory disease primarily affecting peripheral small joints, accompanied by synovial cell proliferation and inflammatory cell infiltration, with a high rate of disability. Traditional treatment methods mainly focus on alleviating symptoms and reducing inflammation, but they are often insufficient for achieving a complete cure (84). UC-MSCs exert immune-modulatory effects by regulating the differentiation, proliferation, and activation of T cell subsets, inhibiting the proliferation and differentiation of B cells, and modulating the maturation of dendritic cells and natural killer cells, thereby suppressing inflammatory responses (85). In a prospective phase I/II clinical trial (NCT01547091), 64 patients with refractory RA, whose disease symptoms were not effectively controlled by DMARDs or NSAIDs and who had persistent symptoms for at least six weeks, received UC-MSCs therapy. During the treatment process, patients initially received 100 mL of saline infusion, followed by intravenous injection of 40 mL of UC-MSCs suspension (2×10^7^ cells/20 mL). One- and three-years post-treatment, patients' blood routine, liver and kidney function, and immunoglobulin levels remained within normal ranges. Compared to pre-treatment values, the levels of ESR, CRP, RF, and anti-CCP were significantly reduced (*p* < 0.05), and the HAQ health index and DAS28 joint function index also significantly decreased (*p* < 0.05). These results indicate that UC-MSCs therapy has long-term efficacy in RA patients (86, 87). Therefore, UC-MSCs demonstrate good safety and preliminary effectiveness in the clinical treatment of RA.

### 3.8 Ankylosing spondylitis

Ankylosing spondylitis (AS) is a chronic inflammatory disease mediated by immune complexes, characterized primarily by spinal bone erosion, new bone formation, and ankylosis. These features lead to severe pain, restricted spinal mobility, and stiffness, with a high incidence and disability rate in clinical practice (88). Patients with AS commonly exhibit a reduction in the number of Treg cells, along with low and dysfunctional B cells (89). In one clinical trial, the therapeutic effects of intravenous infusion of UC-MSCs were evaluated in five patients with AS. Although three patients experienced mild and transient fever within 2 to 6 hours of injection, no serious adverse reactions occurred. After treatment, there was a significant reduction in the Bath Ankylosing Spondylitis Disease Activity Index (BASDAI) and Bath Ankylosing Spondylitis Functional Index (BASFI), with improvements also observed in the functional index. Additionally, three patients showed a reduction in erythrocyte sedimentation rate (ESR), and one patient exhibited a significant decrease in C-reactive protein (CRP) levels, with all patients showing symptom relief (89). Zeng et al. conducted a systematic review and meta-analysis of multiple clinical trials involving UC-MSCs. The control group received treatment with immunosuppressants and biological agents, while the observation group underwent UC-MSCs transplantation. The results indicated that the total effective rate of the observation group was significantly higher than that of the control group (*p* < 0.05). After treatment, the observation group showed significantly lower levels of ESR, intercellular adhesion molecule (ICAM), and serum TNF-α compared to the control group (*p* < 0.05). Moreover, at 1 month, 3 months, and 6 months post-treatment, the pain scores and activity index scores of the observation group were significantly lower than those of the control group (*p* < 0.05) (90). Another study reported the clinical application of UC-MSCs combined with hydroxyapatite (HA) scaffolds for the treatment of vertebral bone defects caused by tuberculosis spondylitis. The study included three patients with tuberculosis spondylitis of the thoracic, thoracolumbar, or lumbar spine, where vertebral collapse exceeded 50%. These patients received a combination transplant of 20 million UC-MSCs and HA particles. Follow-up results showed that 1 month, 3 months, and 6 months post-surgery, the patients’ alkaline phosphatase (ALP) levels gradually increased, and the bone formation rate at the bone defect sites ranged from 50-75%. At 6 months post-surgery, bone formation had increased to 75-100% of the total bone area. The SF-36 questionnaire assessment revealed that patients’ quality of life improved in all domains, with an average total score of 2912.5 ± 116.67 at 6 months (91). These findings suggest that intravenous infusion of UC-MSCs is safe, with good patient tolerance, and can effectively alleviate disease activity and clinical symptoms. Future studies should recruit larger cohorts of AS patients to enable a more systematic evaluation of the therapeutic effects of UC-MSCs.

### 3.9 Allergic rhinitis

Allergic rhinitis (AR) is a common respiratory disorder primarily caused by Type I hypersensitivity reactions in the nasal mucosa. The typical symptoms include episodic sneezing, rhinorrhea, and nasal congestion, which usually persist for more than two days and last for over one hour per day. In severe cases, these symptoms can lead to impaired olfactory function. The sensitization process in AR involves complex interactions among antigen-presenting cells, T cells, and B cells, and is closely associated with the generation of allergen-specific T cells and IgE antibodies (92). Upon re-exposure to allergens, IgE on the surface of mast cells crosslinks and releases allergic mediators such as histamine, triggering nasal symptoms. Within hours, inflammatory cells, especially Th2 cells, eosinophils, and basophils, infiltrate the nasal mucosa, further exacerbating the allergic response. UC-MSCs due to their multilineage differentiation potential, low immunogenicity, and immunomodulatory functions, can migrate to and integrate into the nasal mucosa affected by rhinitis. This process helps improve AR by restoring the Th1/Th2 immune balance and upregulating Treg cell numbers (93, 94). A prospective, open-label, single-center clinical trial is currently evaluating the feasibility of UC-MSCs therapy for AR (NCT05151133). This study plans to recruit 18 patients, dividing them into three groups (6 patients per group), each receiving different doses of UC-MSCs therapy (Group 1: 0.5×10^6^ cells/kg, total volume 100 mL; Group 2: 1.0×10^6^ cells/kg, total volume 100 mL; Group 3: 2.0×10^6^ cells/kg, total volume 100 mL). The primary assessment criteria include the Rhinoconjunctivitis Quality of Life Questionnaire (RQLQ) score, Visual Analog Scale (VAS) score, serum inflammatory markers, nasal secretions, nasal function tests, and nasal endoscopy. Safety indicators such as blood and urine analysis, liver and kidney function, immunological markers, and tumor markers will also be monitored. After the study concludes, researchers will further explore the feasibility of intravenous UC-MSCs infusion for the treatment of moderate to severe persistent AR, based on clinical data and safety evaluations. This will lay the foundation for the next phase of clinical research and provide additional clinical evidence and experience for UC-MSCs therapy in AR patients.

Based on the clinical studies outlined above, UC-MSCs demonstrate a consistent profile of promising efficacy and favorable safety across a spectrum of inflammatory diseases (**Table 2**). The most common adverse reaction to UC-MSC treatment is transient and self-limiting fever, with all studies reporting no serious treatment-related complications or long-term sequelae. UC-MSCs significantly improve disease-specific activity indices, such as CDAI for CD, SLEDAI for SLE, and DAS28 for RA, while also showing survival benefits in critical conditions such as COVID-19 and GVHD. Although protocols vary, repeated dosing (e.g., 2–4 infusions) is typically associated with sustained therapeutic effects, particularly in chronic and severe diseases like GVHD and liver cirrhosis. Both intravenous and local (e.g., intra-articular) administrations are well-tolerated and effective for systemic and localized diseases, respectively. The positive outcomes observed across a range of conditions, from autoimmune diseases (SLE, RA) to degenerative disorders (OA) and acute critical illnesses (COVID-19, aGVHD), highlight the multifunctionality and modular nature of UC-MSCs in immunomodulatory and repair capabilities.

**Table 2:** Selected publications highlighting clinical outcomes of UC-MSCs in targeting inflammatory conditions.

NCT number	Disease	Intervention of UC-MSCs	Result	Adverse reaction	Reference (PMID)
NCT02580695	Knee Osteoarthritis	Intra-articular knee injection; UC-MSCs 20 × 10^6^ diluted in 3 mL of saline solution with 5% of AB Plasma.	Repeated UC-MSC dose strategy led to a favorable safety profile and improved clinical result for the treatment of long-term pain in knee OA patients.	No SAEs, deaths, permanent disability, neoplasia, orseptic arthritis cases were registered during the trial. The most common adverse event related to intra-articular injection was acute synovitis (33% after first injection;44% after second injection).	30592390
NCT02445547	Crohn’s disease (CD)	Intravenous injection; Receive a total of four peripheral intravenous infusions of 1 × 10^6^ cells/kg, with one infusion per week.	Twelve months after treatment, the CDAI, HBI, and corticosteroid dosage had decreased by 62.5 ± 23.2, 3.4 ± 1.2, and 4.2 ± 0.84 mg/day, respectively, in the hUC-MSC group and by 23.6 ± 12.4, 1.2 ± 0.58, and 1.2 ± 0.35 mg/day, respectively, in the control group (*p* < 0.01, *p* < 0.05, and *p* < 0.05 for hUC-MSC vs control, respectively).	Four patients developed a fever after cell infusion. No SAEs were observed.	28873511
NCT01221428	Ulcerative colitis (UC)	Mesenteric artery injection by interventional catheterization; Two groups: The volumes were 50 ml and the average cell number was 3.8 ± 1.6 × 10^7^ (0.5 × 10^6^ cells/kg; range, 2.3 - 4.7 × 10^7^ cells); Other two group: The volumes were 10 ml and the cell number was 1.5 × 10^7^cells.	One month after therapy, 30/36 patients showed good response, and diffuse and deep ulcer formation and severe inflammatory mucosa were improved markedly. During the follow up, the median Mayo score and histology score were decreased while IBDQ scores were significantly improved compared with before treatment (*p* < 0.05).	No evident adverse reactions after MSC infusion in any of the patients, and no chronic side effects or lingering effects appeared during the follow-up period.	27882104
NCT01220492	Decompensated liver cirrhosis (DLC)	Intravenous injection; The treated patients received three times of UC-MSC infusions (Approximately 0.5 × 10^6^ cells/kg were suspended in saline) at 4-week intervals plus conventional treatment that was only used for control group.	During the follow-up check period from 13 to 75th months, there was a significantly higher overall survival rate in the treated group than the control group, while the difference of the hepatocellular carcinoma event-free survival rate between the treated and control groups was not observed during the 75-month follow-up. UC-MSC treatment markedly improved liver function, as indicated by the levels of serum albumin, prothrombin activity, cholinesterase, and total bilirubin during 48 weeks of follow-up.	No significant side effects or treatment-related complications were observed in the hUC-MSC group.	34843069
NCT04355728	COVID-19	Intravenous injection; Subjects in the UC-MSC treatment group received two intravenous infusions (at day 0 and 3) of 100 ± 20 × 10^6^ UC-MSCs; controls received two infusions of vehicle solution.	Inflammatory cytokines were significantly lower in the UC-MSCs treated group at day 6, and their survival was significantly better (91% vs 42%, *p* = 0.015).	No infusion-related SAEs were observed.	33400390
NCT04269525	COVID-19	Intravenous drip; 1 × 10^8^ UC‐MSCs were suspended in 50 mL of normal saline. The patients would receive four rounds of transplantation in total, with one‐day intervals in between. The transplantation was performed about 1.5 hours with a speed of 30‐60 drops per minute.	The oxygenation index was improved after transplantation. The mortality of enrolled patients was 6.25%, whereas the historical mortality rate was 45.4%. The level of cytokines estimated varied in the normal range, the radiological presentations (ground glass opacity) were improved and the lymphocyte count and lymphocyte subsets (CD4 T cells, CD8 T cells and NK cells) count showed recovery after transplantation.	There were no infusion-related or allergic reactions.	33205469
NCT04288102	COVID-19	Intravenous injection; 4.0 × 10^7^ UC-MSCs in a volume of 100 ml/bag; Received three intravenous infusions (at day 0, 3and 6).	The UC-MSCs administration exerted numerical improvement in whole lung lesion volume from baseline to day 28 compared with the placebo (the median difference was −13.31%, 95% CI −29.14%, 2.13%, *p* = 0.080). UC-MSCs significantly reduced the proportions of solid component lesion volume compared with the placebo (median difference: −15.45%; 95% CI −30.82%, −0.39%; P = 0.043). The 6-MWT showed an increased distance in patients treated with UC-MSCs (difference: 27.00 m; 95% CI 0.00, 57.00; *p* = 0.057).	The incidence of adverse events was similar in the two groups.	33568628
NCT00698191	Lupus erythematosus	Intravenous infusion; 1 million cells per kilogram of body weight were administered by intravenous infusion, without adding steroid or other immunosuppressive drugs. Multiple infusions of MSCs were permitted if disease relapsed after the previous infusion, employing the same dose (but not necessarily the same source) of cells for each infusion.	After UC-MSCs treatment, the 5-year overall survival rate was 84% (68/81), with 27% of patients (22/81) achieving complete clinical remission and 7% (6/81) achieving partial clinical remission, for a 5-year disease remission rate of 34% (28/81). Of the 37 patients who achieved clinical remission, 9 subsequently relapsed, for an overall 5-year relapse rate of 24% (9/37). During follow-up, SLE disease activity index, serum albumin, complement C3, peripheral leukocyte and platelet counts, and proteinuria levels continued to improve, suggesting that allogeneic UC-MSCs is safe in patients with SLE and contributes to long-term clinical remission	UC-MSC cells plus DMARDs therapy can be a safe, effective and feasible therapeutic option for RA patients.	29478901
NCT01547091	Rheumatoid arthritis (RA)	Injection immediately; During the treatment, patients were treated with 40 mL hUC-MSC suspension product (2 × 10^7^ cells/20 mL) via intravenous injection immediately after the infusion of 100 mL saline.	1 year and 3 years after hUC-MSC cells treatment, the blood routine, liver and kidney function and immunoglobulin examination showed no abnormalities, which were all in the normal range. The ESR, CRP, RF of 1 year and 3 years after treatment and anti-CCP of 3 years after treatment were detected to be lower than that of pretreatment, which showed significant change (*p* < 0.05). Health index (HAQ) and joint function index (DAS28) decreased 1 year and 3 years after treatment than before treatment (*p* < 0.05).	No serious adverse events or serological or hematological abnormalities observed.	31908418;32664175

SAEs, serious adverse events; OA, osteoarthritis; CDAI, Crohn’s disease activity index; HBI, Harvey Bradshaw index; IBDQ, inflammatory bowel disease questionnaire; DMARDs, disease-modifying anti-rheumatic drugs; ESR, erythrocyte sedimentation rate; CRP, C-Reactive Protein; RF, rheumatoid factor.

## 4 Clinical efficacy assessment and treatment plan optimization

**Figure 4:**
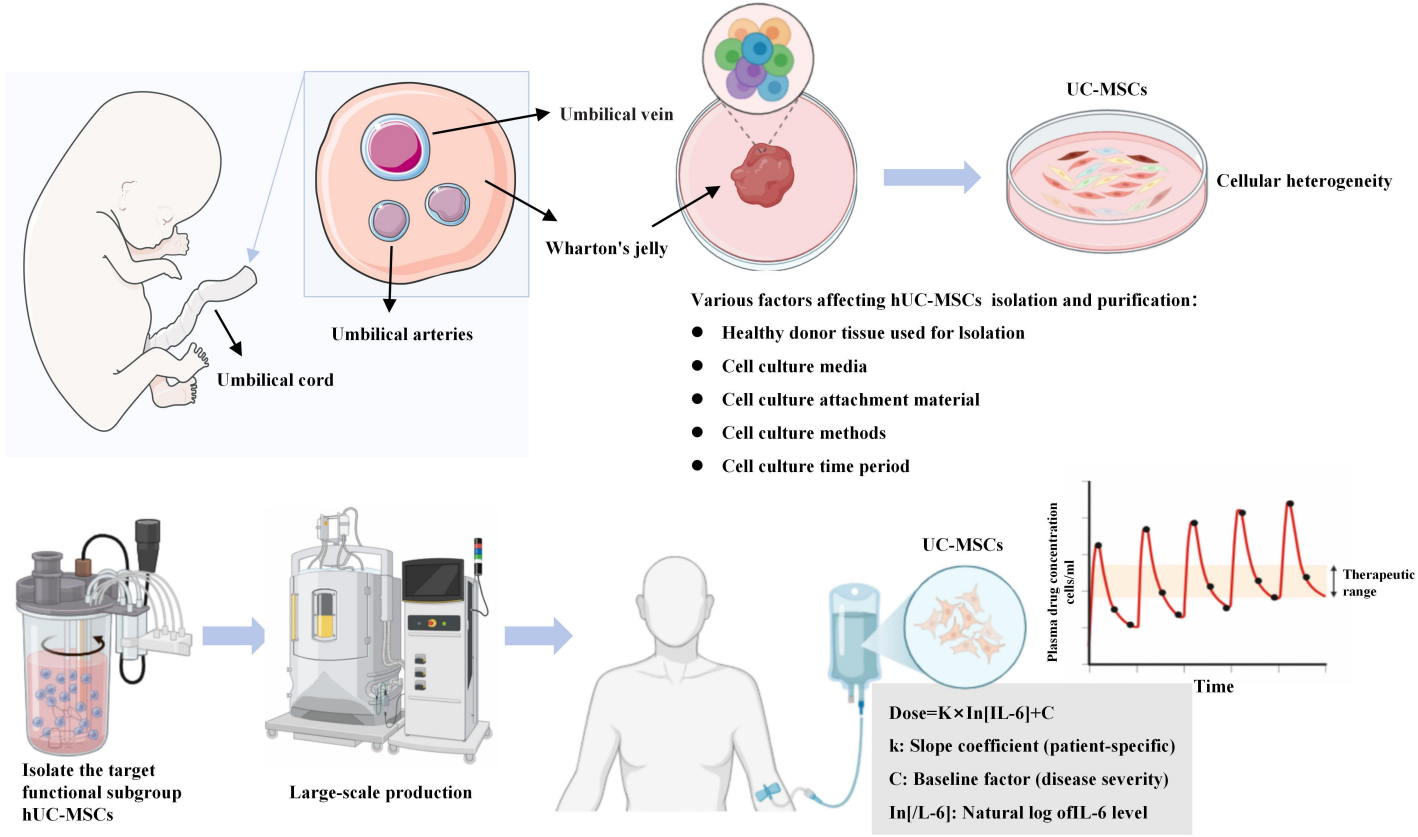
Pharmacokinetics and future challenges in clinical translation. The figure encapsulates translational aspects: A pharmacokinetic profile illustrates viable cell activity over time (dosing, distribution, elimination), indicating dosing frequency (e.g., for GVHD). A dosing algorithm (Dose = k × ln[IL−6] + C) is proposed for personalization. Future challenges (e.g., cell heterogeneity, long-term safety, scalable production) are depicted as missing puzzle pieces.

Due to the extensive biological properties and clinical application potential of UC-MSCs, relevant clinical studies are continuously progressing. Research indicates that the route of administration and dosage of UC-MSCs may be critical factors influencing their therapeutic outcomes (**Figure 4**). Based on existing clinical research literature, this paper summarizes the common administration routes and infusion dosages of UC-MSCs.

### 4.1 Correlation analysis between functional module activity and clinical efficacy

The optimization of UC-MSCs in the treatment of inflammatory diseases deeply depends on understanding the correlation between their functional module activity and clinical efficacy. In this context, a key indicator of UC-MSCs therapeutic efficacy is their capacity to secrete anti-inflammatory cytokines, such as interleukin-10 (IL-10). Recent analyses have demonstrated a strong correlation between IL-10 secretion levels and improvements in clinical outcomes, such as the reduction in the Mayo score among patients with inflammatory bowel disease. A high R² value observed in these studies underscores the significant association between elevated IL-10 secretion and disease amelioration. This robust correlation between functional module activity and clinical endpoints lays a critical biological foundation for designing more effective UC-MSCs-based therapeutic protocols.

Importantly, one of the core elements of optimizing treatment regimens involves determining the appropriate dosage and frequency of administration. For example, several clinical trials utilizing intravenous injection, a common administration route, have employed various dosing schemes (e.g., 1×10^6^ cells/kg weekly for four doses; 0.5×10^6^ cells/kg monthly for two doses; 100 ± 20×10^6^ cells on days 0 and 3 for two doses; 4×10^7^ cells on days 0, 3, and 6 for three doses). These studies have consistently reported significant patient improvements, with overall safety profiles remaining favorable. The most common adverse reactions observed were self-limiting fever (37-38 °C), which typically required no special treatment or only symptomatic management (NCT02445547, NCT01220492, NCT04355728, NCT04288102) (36, 47, 84). Local injections (such as intra-articular injection of 20×10^6^ cells for osteoarthritis) and intrathecal injections (such as 1×10^6^ cells/kg weekly for four doses for spinal cord injury) have similarly demonstrated that specific dosing regimens can effectively improve clinical symptoms and functionality, with related adverse events (e.g., temporary pain post-intra-articular injection (87), occasional headaches and back pain post-intrathecal injection (95) being mostly mild, transient, and manageable (68, 95). These dose-response relationships and the acceptable safety profiles observed in clinical practice further reinforce the critical importance of understanding the correlation between functional module activity (e.g., homing, repair capabilities (96)) and clinical efficacy in guiding safe and effective treatment strategies.

### 4.2 Pharmacokinetic modeling of viable cells and optimizing treatment protocols

Optimizing UC-MSCs-based therapies for inflammatory diseases requires meticulous design of dosing strategies, with the integration of a viable cell pharmacokinetic model being central to this effort. This three-phase model-comprising the initial dosing, distribution, and elimination stages-profoundly contributes to understanding the temporal changes in UC-MSCs activity within the body. It is instrumental in elucidating their clinical behavior and identifying the optimal administration route, dosage, frequency, and intervals. For instance, in the prevention of GVHD, a weekly dosing regimen (~1×10^6^ cells/kg) based on this model has been shown to correlate with sustained immunoregulatory effects and is crucial for maintaining immune balance (97).

Similarly, research on intrathecal injection for spinal cord injury treatment has revealed that patients with incomplete injuries who received multiple treatments (e.g., weekly 1×10^6^ cells/kg for four doses) had a significantly higher success rate (81.25%) compared to those who received a single treatment, with efficacy improving as treatment frequency increased. Notably, the adverse effects reported in this regimen (e.g., headaches, back pain) were mild and transient (95), directly reflecting the necessity of multiple administrations to maintain effective cellular activity, as supported by the pharmacokinetic model and a favorable safety profile.

This pharmacokinetic model aids in determining the optimal dosing intervals and treatment duration, which not only minimizes immune response risks (e.g., by reducing immunogenic accumulation through optimized intervals) but also enhances therapeutic efficacy, allowing healthcare providers to better predict outcomes and customize individual treatment plans. Furthermore, this model facilitates the development of dosing algorithms to interpret nonlinear effects observed in patient outcomes, particularly considering the influence of key inflammatory biomarkers such as IL-6. The proposed algorithm (Dose = k × ln(IL-6) + C) is empirically derived, where the constant C is adjusted according to patient baseline factors (e.g., the need for preventive medication in patients with elevated fasting blood glucose or hypertension, where isopropylamine may replace dexamethasone (86). This logarithmic relationship reflects the diminishing therapeutic returns associated with higher IL-6 levels. Research has confirmed that UC-MSCs significantly lowers IL-6 concentrations, thereby influencing therapeutic outcomes (98, 99).

Numerous studies emphasize the importance of dosing strategies; for instance, Chin et al. observed that higher doses of UC-MSCs in healthy volunteers resulted in more significant immunoregulatory effects compared to lower doses (97), while studies on hepatic arterial injection for cirrhosis indicated that specific doses (e.g., 4×10^8^ cells, two infusions) significantly reduced the incidence of liver failure, with an excellent safety profile (51). Collectively, these findings underscore the necessity of robust dosing algorithms that combine UC-MSCs pharmacokinetic characteristics (which govern distribution, clearance, and required maintenance of activity levels), physiological responses indicated by biomarkers (e.g., IL-6), and individual patient characteristics (e.g., risk factors for adverse reactions). Ongoing clinical trials are assessing the impact of varying UC-MSCs concentrations and dosing protocols on the efficacy and safety of treatments for inflammatory bowel disease and acute liver failure, which will provide crucial insights for further refinement of these precision dosing strategies (100).

## 5 Future directions and emerging paradigms

Despite the promising potential of UC-MSCs in a variety of inflammatory diseases, their clinical translation faces key challenges. Future research should go beyond observational studies and focus on addressing the core issues that will unlock their true therapeutic potential. The therapeutic paradigm of UC-MSCs is undergoing a profound evolution. Future studies should concentrate on cell empowerment and optimization, utilizing gene editing technologies (e.g., CRISPRa) to enhance UC-MSC functions, such as overexpressing key anti-inflammatory factors like IL-10 or PD-L1, thereby generating more robust and sustained immunomodulatory effects post-infusion. Additionally, 3D cultures and bioreactor technologies should be employed to simulate *in vivo* inflammatory microenvironments for educational expansion, activating their anti-inflammatory programs in advance to improve therapeutic efficacy. Notably, UC-MSC-derived exosomes (MSC-Exos) have emerged as a next-generation therapeutic strategy with greater clinical translation potential. As natural, nanoscale vesicles, MSC-Exos carry key functional molecules from their parent cells (such as miRNAs, cytokines, and functional proteins), and due to their phospholipid bilayer structure, they exhibit low immunogenicity and excellent tissue targeting ability (101, 102). Compared to whole-cell therapy, MSC-Exos not only avoid the oncogenic and immune rejection risks associated with cell transplantation but also retain the key functions of promoting tissue repair and immune modulation. In various disease models, including cardiac regeneration and neuroprotection, MSC-Exos have shown similar therapeutic effects to MSCs, such as inhibiting apoptosis, promoting angiogenesis, and alleviating fibrosis (103–105). Their core mechanism lies in their role as efficient carriers of intercellular communication, precisely regulating the biological behavior of recipient cells through the delivery of functional molecules (106). Furthermore, exosomes exhibit more controllable pharmacokinetics, enhanced safety, and repeatable dosing potential, making them suitable for standardized, large-scale production in compliance with drug regulatory requirements (102, 107). The engineering of exosomes into smart drug carriers is a critical area of future research. With continuous advances in pharmaceutical-grade exosome isolation and purification technologies, MSC-Exos-based therapies are expected to gradually supplement or even replace traditional cell therapies, opening new avenues for the clinical management of inflammatory and degenerative diseases (108–110). By surface modification (e.g., incorporating peptides targeting inflammatory sites like VEGFR1) to confer precise homing ability to lesions, and by loading specific anti-inflammatory miRNAs (such as miR-146a-5p) or enzymes (such as extracellular superoxide dismutase), their modular functions can be enhanced to achieve directed delivery and on-demand release for precise treatment.

Biomarker-driven personalized treatment is a key pathway for UC-MSCs to advance toward precision medicine. The core direction for future research is to establish predictive biomarker systems that can address individual variability in UC-MSC efficacy and enable standardized treatments (111). First, by using computational methods such as machine learning, baseline inflammatory markers (e.g., IL-6, TNF-α levels), immune cell subpopulation profiles (e.g., Th17/Treg ratio), and disease-specific biomarkers should be integrated to construct efficacy prediction models for patient stratification (112, 113). For example, identifying subgroups of SLE or RA patients who respond best to UC-MSC therapy. Second, developing cell potency quality control standards based on functional parameters (such as IL-10 secretion ability or exosome production) rather than merely phenotypic markers (CD73+/CD90+/CD105+), to strengthen cell quality monitoring. Through technologies like single-cell RNA sequencing, the subpopulations with the strongest immunosuppressive activity can be identified and used as the gold standard for cell product quality control. Finally, to ensure the reproducibility and safety of UC-MSC therapy, standardized pathways and regulatory frameworks must be established from the laboratory to the clinic. Global standards should be set for donor screening, cell expansion cycles, culture medium components (explicitly free of xenogeneic serum), cryopreservation, and recovery processes (following ISCT guidelines), achieving standardization in manufacturing processes. Prospective clinical trials should optimize treatment protocols to determine the optimal administration windows (e.g., early intervention vs. refractory stages), routes (intravenous, local, or intrathecal), and individualized dosing algorithms (e.g., based on body weight and inflammatory load) (**Figure 4**) (114). Moreover, long-term safety and regulatory frameworks should be established. For example, a national patient registry system should be created to conduct long-term follow-up for patients treated with UC-MSCs, monitoring their immune status and cancer incidence for several years or even decades. Close collaboration with drug regulatory agencies will be necessary to clarify the approval pathways for innovative UC-MSC and MSC-Exos-based therapies.

UC-MSCs and their derivatives represent a modular, multifunctional paradigm of therapy, with the potential to reshape the treatment landscape of inflammatory diseases. Future success will depend on our ability to achieve three key transformations: from natural products to engineered formulations, from empirical drug use to precision medicine, and from exploratory research to standardized clinical practices. Through interdisciplinary collaboration, UC-MSCs are poised to transition from a promising tool to an indispensable pillar in the treatment of inflammatory diseases.
